# Phthalates and Childhood Body Fat: Study Finds No Evidence of Obesogenicity

**DOI:** 10.1289/ehp.124-A78

**Published:** 2016-04-01

**Authors:** Wendee Nicole

**Affiliations:** Wendee Nicole was awarded the inaugural Mongabay Prize for Environmental Reporting in 2013. She writes for *Discover*, *Scientific American*, *National Wildlife*, and other magazines.

A growing body of evidence suggests that environmental “obesogens”—chemicals that alter metabolism, leading to increased fat production and deposition—may be partly responsible for the increasing prevalence of childhood obesity.^1^^,^^2^ A small number of human studies have suggested that certain *ortho-*phthalates may act as obesogens following prenatal exposure.^3^^,^^4^ However, a study published in this issue of *EHP* found not only no association between prenatal phthalate exposures and increased body fat in children, but also that high exposure to di(2-ethylhexyl) phthalate (DEHP) was associated with lower body fat.^5^

Phthalates are used in many consumer products, including toys, food packaging, cosmetics, and pharmaceuticals. The metabolites of these compounds are found almost universally in human urine and have been detected in amniotic fluid. Past human studies have linked early-life phthalate exposures with altered neurological development, childhood allergies, and decreased anogenital distance in baby boys (a marker of feminization).^6^^,^^7^ Several phthalate metabolites exhibit anti-androgenic activity, and there is evidence that some developmental end points vary by sex.^8^

**Figure:**
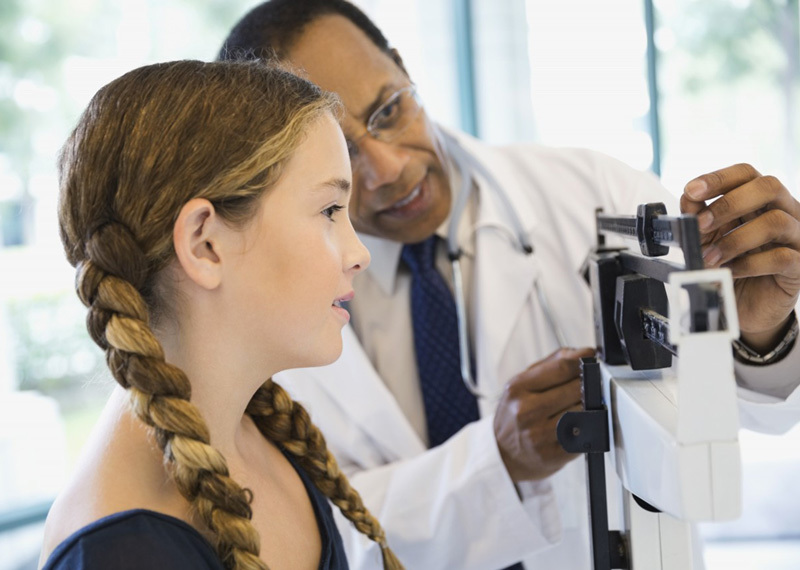
Contrary to earlier evidence suggesting phthalates may act as obesogens, a new study found no association between prenatal phthalate exposure and increased fat mass in childhood. © Hero Images/Corbis

The authors of the current longitudinal, prospective study measured phthalate exposures in pregnant women in their third trimester at Mount Sinai Medical Center in New York. Later, they measured fat mass in 180 of the women’s children at up to three follow-up visits, with the final visit between ages 7 and 9 years. The researchers measured body fat using bioelectrical impedance analysis, in which a very mild electrical signal is sent through the body. Fat resists the signal more strongly than muscle and bone, producing a highly accurate measure of body fat.

The finding that high prenatal DEHP exposure was associated with lower body fat in children runs counter to the hypothesis that phthalates are environmental obesogens. This hypothesis is based in part on evidence that phthalates interact with peroxisome proliferator-activated receptors, which are involved in metabolism.^9^ However, says first author Jessie Buckley, a postdoctoral research associate in the Department of Epidemiology at the University of North Carolina, “This finding is to some extent supported by animal studies of relatively high dose postnatal DEHP exposure that report lower body fat.”

One of the challenges in this area of research is addressing the relevant critical window of exposure. “Most human studies of phthalates and adiposity have assessed postnatal exposures,” Buckley says. “We focused on prenatal exposures because susceptibility to obesity is thought to be ‘programmed’ during fetal development, making the fetus particularly sensitive to environmental exposures that affect fat development and accumulation.”

Leonardo Trasande, an associate professor of pediatrics, environmental medicine, and population health at the New York University School of Medicine, says the study methods are quite strong, but the reliance on a single third-trimester urine sample “gives a somewhat crude measure of pregnancy-wide exposure, given that phthalates have such short half-lives.” Trasande, who was not involved with the study, also notes that the statistically significant association for DEHP and lower body fat was seen only in the highest exposure group, and warns against reading too much into the results. Buckley agrees, saying, “In this small literature, there is still no agreement on what, if any, associations to expect in relation to prenatal phthalate exposure and child growth.”

Joe Braun, an assistant professor of epidemiology at the Brown University School of Public Health, says the study is notable for its use of a novel Bayesian method that allowed the authors to include all the phthalates in the same statistical model and still obtain precise estimates of an association between each phthalate and body fat. “Given the relatively modest sample size of the study, it will be necessary to confirm these findings in other studies,” says Braun, who was not involved with this research. “In addition, future studies will need to examine phthalates as a cumulative exposure.”

## References

[1] Grün F Endocrine-disrupting organotin compounds are potent inducers of adipogenesis in vertebrates. Mol Endocrinol 20 9 2141 2155 2006, doi:10.1210/me.2005-0367#sthash.CXC9SOE8.dpuf 16613991

[2] Chamorro-García R Transgenerational inheritance of increased fat depot size, stem cell reprogramming, and hepatic steatosis elicited by prenatal exposure to the obesogen tributyltin in mice. Environ Health Perspect 121 3 359 366 2013, doi:10.1289/ehp.1205701 23322813 PMC3621201

[3] Valvi D Prenatal phthalate exposure and childhood growth and blood pressure: evidence from the Spanish INMA-Sabadell birth cohort study. Environ Health Perspect 123 10 1022 1029 2015, doi:10.1289/ehp.1408887 25850106 PMC4590754

[4] Buckley JP Prenatal phthalate exposures and body mass index among 4 to 7 year old children: a pooled analysis. Epidemiology, doi: 10.1097/EDE.0000000000000436 [online 6 January 2016] PMC4821741 26745610

[5] Buckley JP Prenatal phthalate exposures and childhood fat mass in a New York City cohort. Environ Health Perspect 124 4 507 513 2016, doi:10.1289/ehp.1509788 26308089 PMC4829985

[6] Swan SH Environmental phthalate exposure in relation to reproductive outcomes and other health endpoints in humans. Environ Res 108 2 177 184 2008, doi:10.1016/j.envres.2008.08.007 18949837 PMC2775531

[7] Braun JM Phthalate exposure and children’s health. Curr Opin Pediatr 25 2 247 254 2013, doi:10.1097/MOP.0b013e32835e1eb6 23429708 PMC3747651

[8] National Research Council. Phthalates and Cumulative Risk Assessment: The Tasks Ahead. Washington, DC:National Academies Press. Available: http://www.nap.edu/catalog/12528/phthalates-and-cumulative-risk-assessment-the-task-ahead [accessed 8 February 2016]

[9] Desvergne B PPAR-mediated activity of phthalates: a link to the obesity epidemic? Mol Cell Endocrinol 304 1–2 43 48, doi:10.1016/j.mce.2009.02.017 19433246

